# Automated Detection and Classification of Oral Squamous Cell Carcinoma Using Deep Neural Networks

**DOI:** 10.3390/diagnostics13050918

**Published:** 2023-02-28

**Authors:** Balasundaram Ananthakrishnan, Ayesha Shaik, Soham Kumar, S. O. Narendran, Khushi Mattu, Muthu Subash Kavitha

**Affiliations:** 1 Centre for Cyber Physical Systems, Vellore Institute of Technology, Chennai 600127, India; 2 School of Computer Science and Engineering, Vellore Institute of Technology, Chennai 600127, India; 3 School of Information and Data Sciences, Nagasaki University, Nagasaki 852-8521, Japan

**Keywords:** medical image analysis, oral cancer, computer vision, feature extraction, CNN

## Abstract

This work aims to classify normal and carcinogenic cells in the oral cavity using two different approaches with an eye towards achieving high accuracy. The first approach extracts local binary patterns and metrics derived from a histogram from the dataset and is fed to several machine-learning models. The second approach uses a combination of neural networks as a backbone feature extractor and a random forest for classification. The results show that information can be learnt effectively from limited training images using these approaches. Some approaches use deep learning algorithms to generate a bounding box that can locate the suspected lesion. Other approaches use handcrafted textural feature extraction techniques and feed the resultant feature vectors to a classification model. The proposed method will extract the features pertaining to the images using pre-trained convolution neural networks (CNN) and train a classification model using the resulting feature vectors. By using the extracted features from a pre-trained CNN model to train a random forest, the problem of requiring a large amount of data to train deep learning models is bypassed. The study selected a dataset consisting of 1224 images, which were divided into two sets with varying resolutions.The performance of the model is calculated based on accuracy, specificity, sensitivity, and the area under curve (AUC). The proposed work is able to produce a highest test accuracy of 96.94% and an AUC of 0.976 using 696 images of 400× magnification and a highest test accuracy of 99.65% and an AUC of 0.9983 using only 528 images of 100× magnification images.

## 1. Introduction

Oral cancer is the sixteenth most prevalent type of cancer in the world (GLOBOCAN 2020). Papua Guinea has the highest age standardized rate of oral cancer cases followed by Bangladesh. India contributes to approximately 30% of the total number of cases in the world (219,772 out of 744,994) [1,2]. Due to rising tobacco and alcohol use, oral cancer incidence rates have been sharply rising in recent years in regions such as Melanesia, South-Central Asia, and Central and Eastern Europe [3]. It has even spread to Australia, New Zealand, and North America, although to a lesser extent. [1] Oral squamous cell carcinoma (OSCC) predominates among all oral cancer cases (84–97%). Inflammatory oral submucous fibrosis, erythroplakia, leukoplakia and proliferative verrucous leukoplakia (PVL) are considered as precursors of OSCC and can indicate the progression of the disease [4]. The buccal mucosa is the most commonly affected area in oral cancer. The effectiveness of remedies in late phase detection is low. Studies show that only 20% of people survive five years after being diagnosed with this disease [5].

Despite being a cancer with high worldwide incidence, oral cancer has received little attention in the field of research [6]. Thus, OSCC has shown high morbidity and mortality among patients of different sexes and age in the past decades. According to the American Joint Committee on Cancer, 70% of oral cancer cases in India are reported to be in advanced stages (stage III-IV). The early detection of metastases plays a crucial role in a patient’s survival, efficiency of diagnosis and chances of relapse [5]. Histopathology using computer-aided techniques can provide promising results in the detection and diagnosis of OSCC.

Medical image analysis is an emerging technology in the era of modern medicine. It focuses on applying computer vision, virtual reality and robotics to analyse biomedical images [7]. It aims to change the traditional healthcare system in an all-round way, helping medical practitioners obtain a quicker and better understanding of any possible disease.

Deep learning is useful in various domains such as computer vision, natural language processing, speech processing, etc. [8]. However, training deep learning models can be computationally expensive and it requires lot of memory because it needs a large amount of labelled data. In medical healthcare, a obtaining large amount of annotated medical data can be expensive [9].

In the previously applied approaches, the images are either taken whole or cropped based on the region of interest (ROI) selected by pathologists. These images are pre-processed and nucleus segmentation is performed (in some). The statistical, morphological and textural features are extracted, and feature size reduced using principal component analysis. The features are then fed into the classifier such as a support vector machine (SVM), logistic regression and decision Tree to predict if the images of OSCC are normal or malignant. Handcrafted feature extraction techniques can give promising results on a small dataset of medical images [10]. However, the extraction process is time consuming and needs a priori knowledge of experts in the domain [11].

The motivation behind the project is to contribute towards automated detection and the classification of oral squamous cells using deep learning, thereby, leading to a faster, cost-effective and more accurate diagnosis with high accuracy. The objective of this work is to provide insights on various ways to efficiently detect carcinogenic cells. A detailed comparison has been made using textural feature extraction techniques (such as local binary patterns, histogram feature extraction) to show that CNN-based feature extraction can effectively improve the detection of OSCC. The proposed work was able to achieve notable accuracy even with a smaller collection of medical images.

## 2. Literature Survey

In the study of textural pattern classification for OSCC, Rahman et al., (2017) employed histogram and gray-level co-occurrence matrix (GLCM) techniques for feature extraction. To select the most relevant features, statistical methods such as t test and principal component analysis (PCA) were used. The images in the dataset were preprocessed by cropping out the background and only keeping the relevant regions. With this approach, the team achieved an accuracy of 89.7% using the t test-generated dataset, and 100% accuracy using the PCA-generated dataset [12].

In a 2020 study, Fua et al., used deep learning algorithms, specifically a cascaded convolutional neural network, to detect oral cavity squamous cell carcinoma from photographic images. They collected images retrospectively from multiple hospitals and augmented the dataset through image processing. They used transfer learning and the receiver operating characteristic (ROC) curve to evaluate the performance of the algorithm. With this method, they achieved an AUC of 0.995, an accuracy of 95.3%, a sensitivity of 97.4% and a specificity of 93.5% [13].

Rahman et al., (2020) in another study proposed a combined method for the classification of OSCC using traditional machine learning techniques. The method achieved the best segmentation of cell nuclei using 40 biopsy slides. They extracted morphological and textural features from 452 hand-cropped cell nuclei and input them into five classifiers: SVM, logistic regression, LDA, k-NN and decision tree. The team achieved an accuracy of 99.78% using a decision tree classifier [14].

Shavlokhova et al., (2021) used a CNN (MobileNet) to detect OSCC using ex vivo fuzzy c-means (FCM) images. The model achieved a specificity of 0.96 and a sensitivity of 0.47 for correct detection and diagnosis. The average processing time was around 30 s and it was found to be dependent on image size. The model performed well in identifying larger malignant areas but struggled to locate smaller or fragmented malignant regions [15].

Ariji et al., (2020) used a deep learning technique called ‘DetectNet’ for the automatic detection of cervical lymph nodes in patients with OSCC in a preliminary study. The training was completed for 1000 epochs and it showed a recall of 90% and 80%, respectively, for metastatic and non-metastatic lymph nodes, and a recall of 73.0% and 52.5%, respectively, on the test dataset [16].

Alabi et al., (2021) conducted a systematic review on the use of machine learning techniques in OSCC. They mainly used artificial neural network (ANN) and SVM techniques and found an accuracy range between 63.4% and 100%. The study suggests that further development is needed to improve explainability, interpretability and external validation before they can be used in clinical practice [17].

Muthu Rama Krishnan et al., (2011) used a hybrid feature extraction paradigm for the automated identification of oral cancer using histopathological images. They extracted textural changes using HOS, LBP and LTE and input them into five classifiers (decision tree, fuzzy, GMM, K-NN, RBPNN) to select the best one. The ensemble model they created, combining texture and HOS features with a fuzzy classifier, achieved 95.7% accuracy, 94.5% sensitivity and 98.8% specificity [18].

Suliman Mohamed Fati et al., (2022) proposed two methods for the early diagnosis of oral squamous cell carcinoma (OSCC) using histopathological images. The first method combined CNN models and an SVM algorithm and achieved superior results in diagnosing OSCC. The second method combines CNN features with colour, texture and shape features extracted using various algorithms, and uses PCA and ANN for diagnosis, resulting in an accuracy of 99.1%, a specificity of 99.61%, a sensitivity of 99.5%, a precision of 99.71% and an AUC of 99.52% [19].

## 3. Proposed System

**Figure 1:**
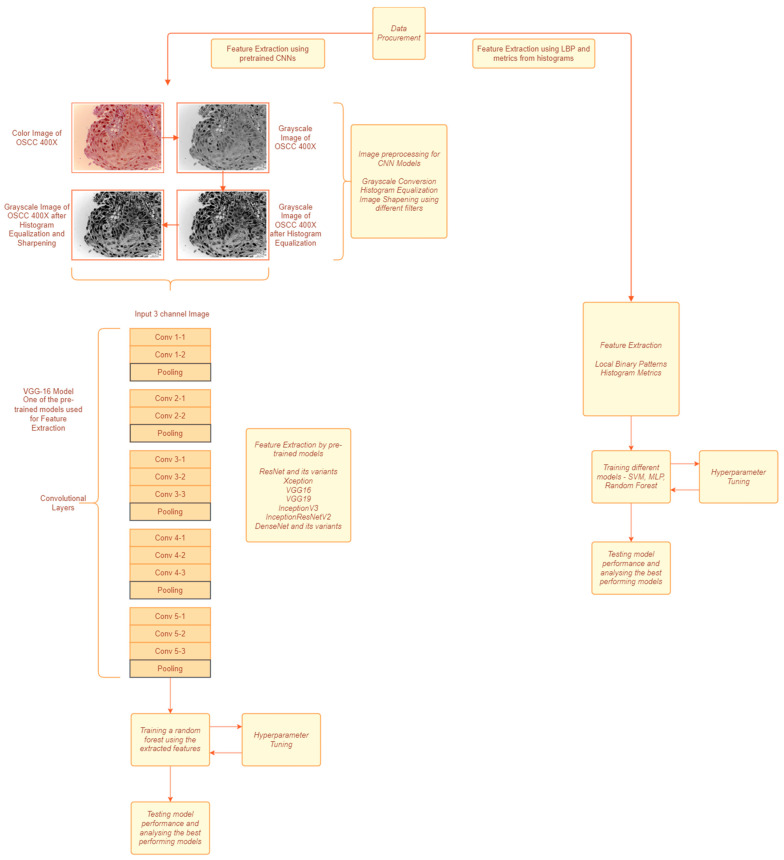
A block diagram of the proposed methodology. The left path is approach used for CNN based feature extraction. The right path is approach used for textural feature extraction.

The data features are extracted using handcrafted techniques in the first approach as shown in Figure 1. For the second approach, the data are first preprocessed and pretrained and CNN models are used to extract the features from the preprocessed images. The features extracted in the first method were fed to various classification models and the best performing model was chosen to predict based on the features extracted from the second method.

### 3.1. Dataset Procurement

The study used H&E-stained punch biopsy slides acquired from the Dr. B. Borooah Cancer Institute and Ayursundra Healthcare in India between October 2016 and November 2017. The tissue sections were from the buccal mucosa, the most common site of oral cancer. The images were taken from patients who visited these organizations for oral biopsy tests. The tissue samples were fixed, dehydrated, cleared and embedded in paraffin wax. Serial sections were formed and stained with H&E [20].

The dataset is divided into two parts with images at 100× and 400× magnification under a Leica DM 750 microscope (model ICC50 HD). The first set has 89 images of normal oral epithelium and 439 images of OSCC and the second set has 201 images of normal oral epithelium and 495 images of OSCC [21].

### 3.2. Handling Class Imbalance

The number of normal images were small compared to the number of OSCC images. Therefore, random images were selected from the normal image dataset, image augmentation was completed on a random rotation, translation and sheer. The normal image dataset was then of the same size as the OSCC image dataset. It improves the generalization and predictability of the model [22,23].

### 3.3. Image Pre-Processing for Pre-Trained Models

**Figure 2:**
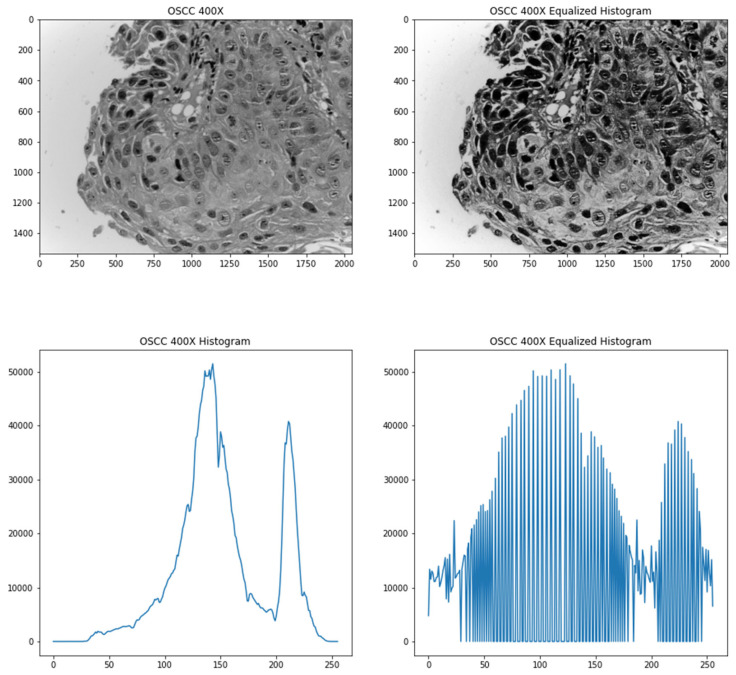
Top-left image shows the original image converted to grey scale; bottom-left image shows the histogram of the original image; top-right image shows the processed image after histogram equalization; bottom-right image shows the histogram of the processed image.

The images are initially converted to greyscale since it resulted in better accuracies, which was found through experimentation. To improve the contrast of the images, a contrast enhancement method known as histogram equalization was used. Histograms are computed by getting a count of the number of pixels with a particular grey level in a greyscale image. Since the images that are being used are 8-bit grayscale images, there are 256 different possible intensities. However, the count of the pixel values is not spread evenly as indicated by the narrow ranges plotted in the bottom left of the image. Therefore, to make it more evenly distributed, a pixel value is mapped to another value based on the cumulative distribution of the pixel intensities. This paper used adaptive histogram based equalization (AHE) which preserves image contrast [24]. The histogram equalization formula is shown in (1).

(1) $$\;h\left(v\right)=round\left(\frac{cdf\left(v\right)-cdf_{min}}{\left(M\times N\right)-\;cdf_{min}}\times \left(L-1\right)\right)$$

where *h*(*v*) is the resultant pixel value, *cdf* or cumulative distribution function is the sum of the all the counts of pixel intensities to that pixel value, *cdf*(*v*) is the cumulative distribution function of *v*, *cdf_min_* denotes the minimum non-zero value of the *cdf*, (*M* × *N*) is the total number of pixels that the image has and *L* is the number of grey-levels in an image (256 for 8-bit grayscale images). Figure 2 shows an image and its respective histogram equalized version with its respective histogram plots.

Analysing the images manually, there were a number of images that were blurry. Having blurred images resulted in poorer feature extraction. To tackle that problem, a sharpening kernel is used to sharpen the images. Enhancing the edges of objects and modifying the contrast and shadow characteristics are performed with sharpening filters. These filters can be used as edge detectors when combined with a threshold. Sharpening or high-pass filters are incredibly sensitive to noise while letting high frequencies pass and reducing lower frequencies [25]. When editing many different types of photographs, sharpening is essential since it helps to highlight the features. When sharpening an image, it should be kept in mind that sharpening should be the final step in a filtering pipeline because it is particularly output-specific. Sharpening filters are extremely noise-sensitive. If necessary, noise reduction should always be used first. Since sharpening can produce undesirable edge effects or increase image noise, choosing the right radius is essential for obtaining acceptable results.

The sharpening kernel that is used is given. It is convolved with the image matrix to obtain the sharpened image.

$$\left[\begin{array}{ccc} 0 & -1 & 0\\ -1 & 5 & -1\\ 0 & -1 & 0 \end{array}\right]$$

**Figure 3:**
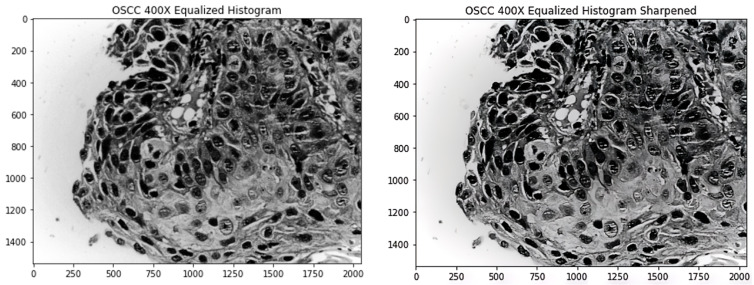
Left image shows the original processed by histogram equalization; right image shows the sharpened left image.

Figure 3 shows an image after it is processed using the equalized histogram technique and the new image after sharpening is applied on the image.

### 3.4. Feature Extraction by Pre-Trained Models

CNNs are one of the most popular neural network architectures that are used in a variety of domains ranging from surveillance to healthcare [26]. These networks contain convolutional and pooling layers in succession which learns features pertaining to the images while training. These networks contain several layers of convolutional and pooling layers wherein the lower level layers learn lower level details of the images (such as lines, edges) and the deeper level layers learn higher level details of the images (such as an eye or a nose in the case of face detection).

Several different pretrained CNNs are used, namely, ResNet and its variants, Xception, VGG16, VGG19, InceptionV3, DenseNet and its variants. These networks are trained on a large dataset known as “imagenet” that contains 1000 different classes. The weights learned by the networks from that dataset can be reused in another domain. This concept of transferring knowledge gained in one domain to an application in another domain is known as transfer learning.

VGG uses small filters (3 × 3 convolution filters) with periodic pooling filter and its depth is 16-19 layers. It has more nonlinearities and fewer parameters. The fully connected layers of VGG can be used as a good feature representation to extract features from other data [27]. However, total memory usage of this network is heavy.

Inception focuses on improving computational efficiency using efficient “inception” modules. It has a depth of 22 layers. It has no fully connected layers which greatly reduces the number of parameters. Inception modules apply parallel filter operations on an input from the previous layer, concatenate all the results and send them to the next layer. Thus, it preserves special dimensions while reducing feature depth using “bottleneck” layers that use 1 × 1 convolutions [28].

The ResNet architecture is designed to address the issue of deep networks underperforming shallower networks. It utilizes a “residual” structure, which aims to fit a residual mapping *F*(*x*) instead of attempting to directly fit the desired underlying mapping *H*(*x*) (He at al., 2015). The residual mapping is defined as the difference between *H*(*x*) and the identity mapping of the input “x”. This allows the network to learn residuals or the adjustments needed to improve the performance of the identity mapping. The architecture is composed of layers that range from 50 to 152, with each residual block containing two 3 × 3 convolutional layers. Additionally, the number of filters is periodically doubled, and the spatial resolution is reduced by a stride value of 2. Similar to the inception architecture, it does not contain fully connected layers, and “bottleneck” layers are used to reduce computational complexity [29].

The DenseNet architecture utilizes “dense” blocks, in which each layer is connected to all other layers in a feed-forward manner. This approach addresses the vanishing gradient problem and strengthens feature propagation, enabling feature reuse. This leads to improved performance compared to ResNet, while using fewer parameters. However, the dense connectivity can decrease the computation-efficiency and parameter-efficiency of the network, and make it more susceptible to overfitting [30]. Feature extraction using CNNs that were previously trained on other large image datasets with hundreds of classes is used. These features are fed as an input to a random forest, which is trained on the extracted features.

### 3.5. Textural Feature Extraction

#### 3.5.1. LBP Feature Extraction

**Figure 4:**
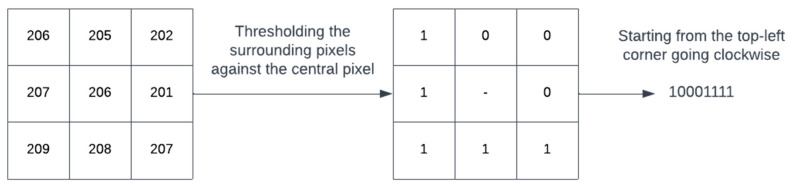
An example to demonstrate LBP mechanism.

Figure 4 shows the working of a simple local binary patterns (LBP). LBP is an image descriptor that is used to describe textures in images. Using the descriptor, it is possible to distinguish between different textures found in images. A window surrounding a pixel is considered. Traversing the pixels in the window one by one, it assigns a binary value of ‘1’ to a pixel if its value is higher than the central pixel, otherwise it assigns a value of ‘0’ as shown in Figure 4. This results in a binary pattern that represents the texture of the image. Due to their robustness to noise, illumination changes, rotation and its computational efficiency, LBP operators have been widely used in various fields such as biomedical image analysis, facial recognition and object detection [31].

The resulting binary pattern is then converted to decimal, and a histogram is computed across the image resulting in a 256-feature vector. Some implementations such as skimage result in a 26-feature vector. The vectors can be used to train machine learning models to classify the image into different classes in a dataset.

#### 3.5.2. Histogram Metrics Feature Extraction

As Rahman et al. [12] have discussed in their approach, statistical metrics of the grey-level histogram of images can be used as a way to describe texture in images. Statistical measures such as mean, variance, skewness, kurtosis and entropy of the histograms of images are taken as feature vectors. These features vectors are fed into the model separately. Since these feature vectors weren’t able to produce satisfactory results standalone, the models were trained by combining the histogram metrics and the LBP features.

### 3.6. Training a Classifier

#### 3.6.1. SVM

A support vector machine (SVM) is a supervised machine learning algorithm that is used for classification and regression tasks. The SVM algorithm finds an N-dimensional hyperplane, which is a decision surface that separates the data into different classes. The N dimensions refer to the number of features present in the dataset. To achieve this, the algorithm maximizes the distance between the data points and the hyperplane, known as the margin. The hinge loss function is often used to optimize the distance and find the best hyperplane that separates the data. The support vectors are the data points that are closest to the decision boundary and have the greatest impact on the position of the hyperplane.

Support vector machines are effective in high-dimensional spaces. Even when there are more dimensions than samples, the algorithm still proves to be effective. Hinge loss (2) is used to calculate the cost function. The cost function is minimized to obtain the best fit model.

(2) $$c\left(x,y,f\left(x\right)\right)={\left(1-y*f\left(x\right)\right)}_{+}$$

The input data can be transformed using different kernel functions *f*(*x*). These kernel functions can aid in transforming non-separable data to separable data.

The kernels that are used are linear, polynomial, RBF (radial basis function) and sigmoid.

During the training phase of the SVM model, when the model is able to correctly predict the class of a data point, the parameters are updated using the gradient from the regularization term. λ as shown in (3).

(3) $$\omega =\omega -\alpha \;\cdot \;\left(2\lambda \omega \right)$$

When the model misclassifies a data point, the parameters are updated using not only the regularization parameter but also the loss as shown in Equation (4).

(4) $$\omega =\omega +\alpha .\;\left(y_{i}\;\cdot \;x_{i}-2\lambda \omega \right)$$

The decision function can be provided with a variety of kernel functions as mentioned before. While training the SVM models, the parameters such as the kernel, gamma and shape of the decision function were changed to different values and the one that would give the highest accuracy was obtained.

#### 3.6.2. Multi-Layer Perceptron

A multi-layer perceptron (MLP) is a type of neural network that is composed of interconnected nodes, also known as neurons. These neurons take a weighted sum of the input, pass it through an activation function and produce an output. The network is composed of an input layer, one or more hidden layers and an output layer. The input layer has the same number of nodes as the number of features in a sample. The hidden layers have several layers stacked one after the other, each containing multiple nodes. The output layer contains one node for binary classification or the same number of nodes as the number of classes in the dataset. The connections between the layers are determined by the weights of the connections. The weighted sum, which is given as an input to each node, is the dot product of the weights of the connections and the output from the previous layer. Each node in the MLP uses an activation function, commonly a sigmoid function, which maps the input to a value between 0 and 1 as shown in (5).

(5) $$sigmoid\left(x\right)=\frac{1}{\left(1+exp\left(-x\right)\right)}$$

During the training phase of a multi-layer perceptron (MLP) model, the parameters of the model are initially set to random values. The weighted sum of the input is calculated using these randomly initialized weights and then passed through an activation function. This process is repeated at every node in every layer and is known as forward propagation. The loss function is then calculated and the partial derivatives of the loss function with respect to the model parameters are computed. These derivatives are used to update the parameters in a process called backward propagation. [32] There are several optimization functions that can be used to find the optimal set of parameters, such as stochastic gradient descent and LBFGS. The loss function, as shown in (6), may also include a regularization term that helps to reduce the model parameters to prevent overfitting [33,34].

(6) $$C\left(y,\;w,\;X,\;b\right)=\frac{1}{N}\;\overset{N}{\underset{i=1}{\sum }}{\left(y_{i}-max\left(0,\;w\;\cdot \;X_{i}+b\right)\right)}^{2}$$

#### 3.6.3. Random Forest

A random forest model is trained on each of the feature sets that are output from the pretrained CNN models. Random forest is an ensemble learning method; it contains a large number of decision trees and averages over the trees to classify the input data. The decision trees are trained on bootstrapped data, i.e., a subset of the dataset is sampled randomly and duplicated to match the original dataset size unless mentioned otherwise. They are also trained on a subset of features from the original dataset. Therefore, the trees are as uncorrelated as possible [35].

The decision trees that form the random forest consist of the root node and internal nodes that take a decision at each node resulting in the classification of the input vector at the leaf nodes. There are several popular algorithms to train and build the decision trees such as Iterative Dichotomiser 3 (ID3) and classification and regression tree (CART). The splitting criteria on each node is governed by Gini impurity or entropy. In the random forests that were built for the problem, Gini impurity was used and its formula is shown in (7) [36].

(7) $$Gini=1-\overset{n}{\underset{i=1}{\sum }}{\left(p_{i}\right)}^{2}$$

where *p_i_* represents the proportion of the sample belonging to a class n for a particular node.

Since CART is nonparametric, it does not depend on data coming from a specific kind of distribution.

Outliers in the input variables have little impact on CART. Decision trees can “expand” if stopping rules are relaxed and the tree can subsequently be pruned down to its ideal size. This strategy reduces the likelihood that the crucial data set structure would be missed due to premature stopping. To more effectively determine the goodness of fit, CART combines testing used test data sets and cross-validation. The CART algorithm is capable of using the same variables several times across the tree. This skill can reveal intricate relationships between groups of variables. To choose the input set of variables, CART can be used in conjunction with other prediction techniques. However, CART models are biased towards features with many outcomes or levels and can have a bias towards features that have a high proportion of the majority class. Therefore, it is important to pay attention to the distribution of the classes in the data and balance them, if necessary.

### 3.7. Hyperparameter Tuning

#### 3.7.1. Textural Feature Learning

While training the SVM model, the hyperparameters kernel, gamma and decision_function_shape are explored. Kernel acts as a tuning parameter here and helps to improve the accuracy. Gamma is basically the coefficient of the kernel. Decision_function_shape is used to decide whether to choose a one-vs-rest shape or a one-vs-one shape. For the 400× images, the highest accuracy achieved is with the kernel parameter ‘rbf’, the gamma parameter ‘auto’ and the decision_function_shape ‘ovo’. The accuracy was 84.4%. For the 100× images, the highest accuracy achieved was 90.34%.

For MLP, the activation and solver hyperparameters are explored. The activation parameter is the activation function for the hidden layer and the solver is for optimizing weight. For 400× images, the highest accuracy achieved was with activation ‘relu’ and solver ‘adam’. The accuracy was 90.83%. For 100× images, the highest accuracy achieved was 95.52%.

In the random forest model, the hyperparameters, criterion and max_features are explored. The criterion is used to measure the quality of a split and max_features is used to decide the number of features to be considered. For 400× images, the highest accuracy is achieved with criterion ‘gini’ and max_features ‘sqrt’. The accuracy was 94.19%. For 400× images, the highest accuracy of 97.59% was achieved.

#### 3.7.2. CNN-Extracted Feature Learning

The random forest model is fine-tuned using a grid search. In a grid search, a user specifies the hyperparameters to be searched over and their possible values. The grid search algorithm then systematically trains the model for each combination of hyperparameters and evaluates the model’s performance using cross-validation. It returns the model parameters with the best accuracy. The hyperparameters and their ranges taken for exploration are as follows:

n_estimators—[100, 200, …, 1000]

max_depth—[10, 20, …, 100]

min_samples_split—[2, 5, 10]

min_samples_leaf—[1, 2, 4]

boot_strap—[True, False]

‘n_estimators’ refers to the number of trees in the forest and ‘max_depth’ refers to the maximum depth of the tree. If there are none, then the nodes are expanded until all the leaves contain less than min_samples_split samples. To avoid overfitting, the bound is kept from 10 to 100. The term ‘min_samples_split’ refers to the minimum number of samples required to split an internal node while ‘min_samples_leaf’ refers to the minimum number of samples required to be at a leaf node. These two hyperparameters play an important role in ensuring the good fit of the model. Setting ‘min_samples_split’ to a large value will result in more compact trees, while setting it to a small value will result in larger trees with more splits. The minimum integral value is 2. Setting ‘min_samples_leaf’ to a large value will result in more compact trees with fewer leaf nodes, while setting it to a small value will result in larger trees with more leaf nodes. The value ranges chosen are small to increase diversity and reduce variance by allowing the creation of more trees. The term ‘boot_strap’ means creating multiple subsets of the training data and training the trees of the random forest model. The ‘bootstrap’ value of True means sampling will be with the replacement and False means without. The highest accuracy was achieved for the hyperparameter values ‘n_estimators’ = 300, ‘max_depth’ = 10, ’min_samples_split’ = 6 and ‘bootstrap’ = False, while ‘max_features’ = ‘sqrt’ and ‘criterion’ = ‘gini’ are kept same and ‘min_samples_leaf’ has a default value of 2.

## 4. Experimental Results and Discussion

### 4.1. Tools Used

The experiment was carried out in the Colab environment. GPU in the Colab environment (NVIDIA Tesla K80) was used to obtain the features from the pretrained models. The cv2 library is used for converting the images to greyscale, histogram equalization, resizing, computing the histograms and sharpening the images. The Keras API in TensorFlow is used to initialize the pretrained models and to obtain the feature vectors for the input dataset. The Scikit-Learn library is used for initialising and training random forests. The NumPy library is used for calculating the mean, variance, skewness, kurtosis and entropy of vectors derived from computed histograms.

### 4.2. Model Evaluation Metrics

To measure and compare the models’ performances, the following metrics are used: accuracy, sensitivity, specificity. In the Equations (8)–(10), the terms TP, FP, TN, FN stand for true positive, false positive, true negative and false negative, respectively. The TP, FP, TN, FN values are calculated by comparing the model’s predictions against the ground truth. The ground truth is the class that a particular image belongs to in the dataset. The TP, FP, TN and FN are calculated and the confusion matrix is shown in Table 1.

*Accuracy* [37] is the number of testing samples accurately classified in the entire testing set. In other words, it is the probability that the model predicts the condition of the patient accurately (positive or negative).

(8) $$\;Accuracy=\frac{TP+TN}{TP+TN+FP+FN}\times 100\%$$

*Sensitivity* is the number of testing samples accurately classified as positive in all the samples with the ground truth positive. It is also known as the true positive rate (TPR). In other words, it is the probability that a patient who has a disease will be predicted by the model that they contain the disease.

(9) $$\;Sensitivity=\frac{TP}{TP+FN}\times 100\%$$

*Sensitivity* is extremely important for any medical application. If the models are used in the medical domain, it cannot afford to have false negatives. The false positives can always be further tested and confirmed by another method such as a biopsy. However, a model that predicts positive always will have a sensitivity of 100%, therefore, it is also important to have a high specificity.

*Specificity* is the number of testing samples accurately classified as negative in all the samples with the ground truth negative. It is also known as the true negative rate (TNR). In other words, it is the probability that for a patient who does not have the disease the model predicts that they do not have the disease.

(10) $$\;Specificity=\frac{TN}{TN+FP}\times 100\%$$

If a model has high specificity and it predicts that a particular patient is positive, then there is a high chance of that patient contains that disease.

The area under the receiver operating characteristics (AUROC) [38] is a metric that can be used to evaluate classification models. The TPR or *specificity* is plotted against the false positive rate (FPR) or *sensitivity*. A perfect classification model will have an AUROC of 1.

Classification models output a probability that a sample belongs to a particular class and the probability values are turned into discrete numbers (0 or 1 in case of binary classification) based on a threshold parameter T. In ROC curves, the threshold parameter T is varied and the resulting TPR and FPR values are plotted in the graph. Analysing the ROC curves helps in selecting the model with a particular classification threshold based on the requirements of the problem by trading off between sensitivity and specificity. If the problem necessitates a model with high sensitivity, then the ROC curve is analysed and a model can be selected with a particular threshold from it.

### 4.3. Performance Analysis

The images are preprocessed initially. The images are converted to greyscale, the contrast is enhanced by histogram equalization and it is sharpened. A total of 31 parameters were obtained on applying LBP and extracting histogram features from the images. PCA was applied, keeping a variance of 0.99. The data are split into a train-test ratio of 0.67:0.33. Then, the features of training data are fed into the different classification models and the results are observed based on predictions of testing data.

Table 2 lists the performance of different classifiers on textural and histogram features of 400× images. It can be observed that random forest classifier shows a highest accuracy of 94.19%, a specificity of 94.44% and an AUC of 0.9834. Multilayer perceptron shows a highest sensitivity of 97.53%.

**Figure 5:**
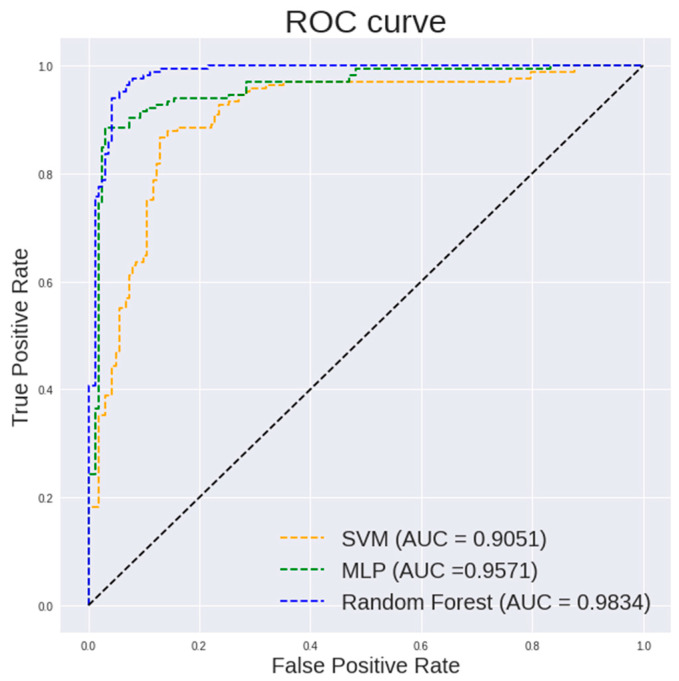
ROC curve of different classification models on textural and histogram features extracted from 400× images.

**Figure 6:**
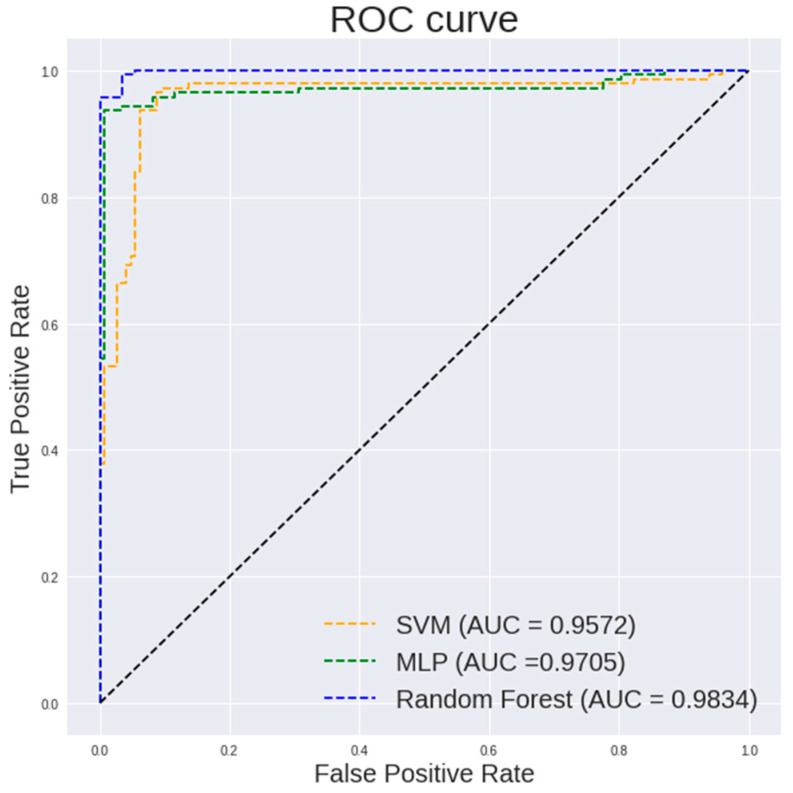
ROC curve of different classification models on textural and histogram features extracted from 100× images.

Figure 5 and Figure 6 show the ROC curve pertaining to the different classification models on textural and histogram features extracted from 400× images and 100× images, respectively. The ROC curve is an important indicator of how well the model is able to perform an effective classification at various thresholds. It is desired to have the curve positioned in the top left corner of the ROC plot. It is observed in Figure 5 that a random forest shows a steeper ROC curve than the other models on textural and histogram features extracted from 400× images which indicates a good performance of the model.

Table 3 highlights the performance of different classifiers on the textual and histogram features of 100× images. It can be observed that a random forest classifier shows a highest accuracy of 97.59%, a sensitivity of 100%, a specificity of 95.1% and an AUC of 0.9984.

It is observed in Figure 5 that a random forest shows a steeper ROC curve than the other models on the textural and histogram features extracted from 100× images which indicates a good performance of the model.

The pretrained models take an input image of size 300 × 300. Table 4 lists the feature vector sizes for an input image from various CNN models to the random forest classifier. These vectors are flattened before training the random forest. The data is split into a train-test ratio of 0.67:0.33. A random forest with 100 estimators or decision trees is initialized and is trained on the flattened vectors of training. The models are tested against the testing dataset. The various pretrained models that were used for feature extraction from 100× magnification images and 400× magnification images and their respective performances were fed to a random forest.

**Figure 7:**
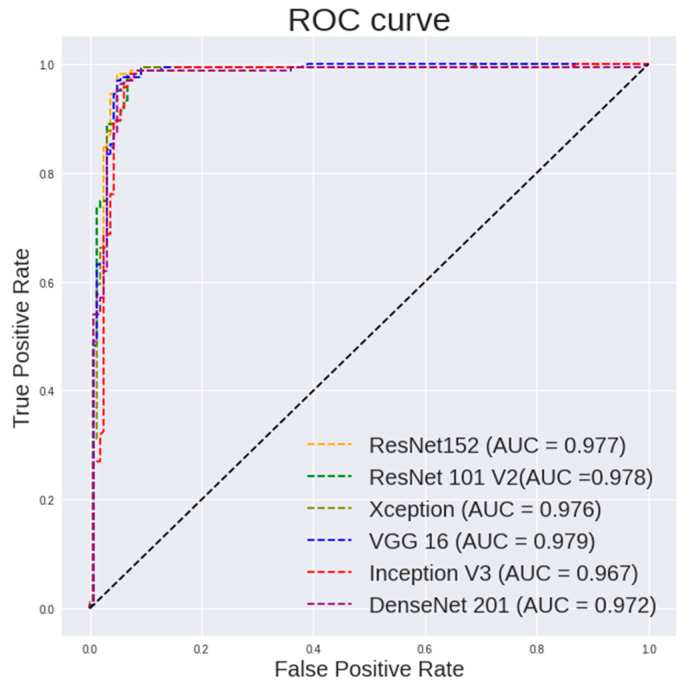
ROC curve for different CNN models with random forest classifier for 400× images classification.

**Figure 8:**
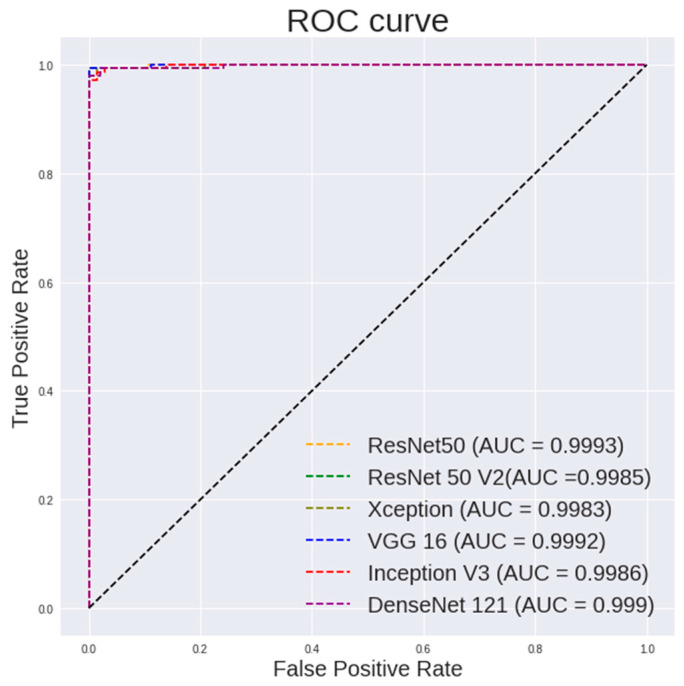
ROC curve for different CNN models with random forest classifier for 100× images classification.

Table 5 highlights the performance of the random forest using features extracted from different pretrained models on 400× images. It can be observed that ResNet101 shows a highest accuracy of 96.94%, a sensitivity of 98.77% and a specificity of 95.12%. ResNet50 shows a highest specificity of 97.53%. VGG16 shows a highest AUC of 0.979. Figure 7 and Figure 8 show the ROC curve pertaining to the different CNN models with the random forest classifier for 400× images and 100× images, respectively.

It is observed in Figure 7 that the random forest shows a steeper ROC curve on the features extracted using ResNet models from 400× images than other CNN models, which indicates good performances of the ResNet models as a feature extractor.

Table 6 lists the performance of the random forest using features extracted from different pretrained models on 100× images. It can be observed that VGG19 shows a highest accuracy of 99.65%, a sensitivity of 99.3% and a specificity of 100%. ResNet50 shows a highest AUC of 0.9993.

It is observed in Figure 8 that the random forest shows a steeper ROC curve on the features extracted using VGG models from 100 × images than other CNN models, which indicates good performances of the VGG models as a feature extractor.

### 4.4. Comparing Proposed Work with Contemporary Work

Table 7 clearly shows how the proposed study has been able to achieve a higher AUC and comparable accuracy without the effort of choosing and extracting specific features from the image. The networks extract the required features while training. It has the capability to capture hidden features from images. An equalized histogram and sharpening enhance the contrast of the image and make the features in the image more prominent. Since the CNN models chosen are already pretrained over a large dataset, the necessity to train these networks on the training dataset is bypassed and, therefore, it can easily extract features from the given images using these models. ANN is a computationally expensive algorithm and requires a large amount of data to effectively learn. Unlike a decision tree, every tree in a random forest is grown without any pruning. Random samples are taken from the dataset and each random forest tree is trained on them, and at each node, a random set of features are considered for splitting. Both mechanisms create diversity among the trees. The randomness and voting mechanisms in random forests elegantly solve the overfitting problem.

## 5. Conclusions

This paper has explored various feature extraction and image preprocessing techniques that will help in the classification of images between normal tissue and malignant OSCC. Two different approaches towards the problem were assessed. Firstly, texture features such as metrics from a histogram and local binary patterns were explored as feature extraction techniques and several machine learning models were trained on the extracted features. For 100× and 400× images, the random forest classifier showed the highest accuracy of 94.19% and 97.59%, respectively. Secondly, several preprocessing techniques (greyscale conversion, histogram equalization and sharpening) were combined and several popular pretrained CNN architectures were explored for feature extraction; their results when combined with random forests were collected and compared. For 400× magnification images, ResNet101 and random forest achieved an accuracy of 96.94%. For 100× magnification images, VGG19 and random forest achieved an accuracy of 99.65%. Thus, it supports the fact that extracting features from pretrained models such as VGGNets and ResNets perform better than extracting features separately. Machine learning models such as random forests used for image classification perform better only when the image dataset is small. In future, a different dataset with samples from different centres and a higher number of samples can be used to build deep learning models that can perform better. Comparison of the models against human experts in the field is needed if the system is to be deployed in the real world.

## Figures and Tables

**Table 1:** Confusion matrix to evaluate classification results.

	Ground Truth—Positive	Ground Truth—Negative
Predicted Positive	True Positive	False Positive
Predicted Negative	False Negative	True Negative

**Table 2:** Performance of different classifiers on textural and histogram features of 400× images.

Model	Accuracy (in %)	Sensitivity (in %)	Specificity (in %)	AUC
Support Vector Machine	84.4	87.65	81.21	0.9051
Multi-Layer Perceptron	90.83	**97.53**	84.24	0.9571
Random Forest	**94.19**	95.68	**94.44**	**0.9834**

**Table 3:** Performance of different classifiers on textural and histogram features of 100× images.

Model	Accuracy (in %)	Sensitivity (in %)	Specificity (in %)	AUC
Support Vector Machine	90.34	93.88	86.71	0.9572
Multi-Layer Perceptron	95.52	99.32	91.61	0.9705
Random Forest	**97.59**	**100**	**95.1**	**0.9984**

**Table 4:** Comparison of input sizes from various CNN models to the random forest classifier.

Model	Feature Vector Shape Fed into Random Forest
ResNet50	(10 × 10 × 2048)
ResNet101	(10 × 10 × 2048)
ResNet152	(10 × 10 × 2048)
ResNet50V2	(10 × 10 × 2048)
ResNet101V2	(10 × 10 × 2048)
ResNet152V2	(10 × 10 × 2048)
Xception	(10 × 10 × 512)
VGG16	(9 × 9 × 512)
VGG19	(9 × 9 × 512)
InceptionV3	(8 × 8 × 2048)
InceptionResNetV2	(8 × 8 × 1536)
DenseNet201	(9 × 9 × 1920)
DenseNet121	(9 × 9 × 1024)
DenseNet169	(9 × 9 × 1664)

**Table 5:** Classification performance of different models on 400× images.

Model + Random Forest	Accuracy (in %)	Sensitivity (in %)	Specificity (in %)	AUC
ResNet50	96.33	96.93	95.73	0.975
ResNet101	**96.94**	**98.77**	**95.12**	**0.976**
ResNet152	96.33	97.55	95.12	0.977
ResNet50V2	94.8	96.93	92.68	0.971
ResNet101V2	92.66	91.41	93.9	0.978
ResNet152V2	94.19	93.25	95.12	0.97
Xception	92.35	88.96	95.73	0.976
VGG16	94.8	97.55	92.07	0.979
VGG19	95.41	98.16	92.68	0.973
InceptionV3	93.58	93.25	93.9	0.968
InceptionResNetV2	90.21	87.12	93.29	0.959
DenseNet201	95.41	96.32	94.51	0.972
DenseNet121	94.8	96.32	93.29	0.972
DenseNet169	94.5	95.71	93.29	0.969

**Table 6:** Classification performance of different models on 100× images.

Model + Random Forest	Accuracy (in %)	Sensitivity (in %)	Specificity (in %)	AUC
ResNet50	99.3	98.59	100	0.9993
ResNet101	99.3	98.59	100	0.9992
ResNet152	99.3	98.59	100	0.9992
ResNet50V2	98.26	96.48	100	0.9985
ResNet101V2	98.95	97.89	100	0.9983
ResNet152V2	98.95	97.89	100	0.9981
Xception	98.61	98.59	98.62	0.9983
VGG16	98.95	97.89	100	0.9992
VGG19	**99.65**	**99.3**	**100**	**0.9983**
InceptionV3	97.56	95.07	100	0.9986
InceptionResNetV2	98.61	97.18	100	0.9982
DenseNet201	97.91	95.77	100	0.9971
DenseNet121	99.3	98.59	100	0.999
DenseNet169	98.61	97.18	100	0.998

**Table 7:** Comparison of results with the proposed study and the previous works on histopathological OSCC images.

Topic	Image Dataset Size Taken	Test Data	Image Features	Classifier	Accuracy	Auc
**Study of morphological and textural features for classification of OSCC by traditional machine learning techniques [12]**	334 OSCC, 118 Normal images	5-folds out of which 1-fold was for testing and rest for training. Average of all 5 test results taken	Morphological Textural Combined	Decision Tree SVM Decision Tree	99.3 99.2 99.78	0.99
**Early Diagnosis of Oral Squamous Cell Carcinoma Based on Histopathological Images Using Deep and Hybrid Learning Approaches [19]**	2698 OSCC, 2494 Normal images	20% of data taken for testing and rest for training	ResNet-18 + DWT, LBP, FCH, and GLCM feature extractions	ANN	99.3	0.9939
**Proposed work**	495 OSCC, 201 Normal Images (400× magnified images) 439 OSCC, 89 Normal images (100× magnified images)	33% of data taken for testing and rest for training 33% of data taken for testing and rest for training	Equalized Histogram + Sharpen+ Features extracted using pre trained weights of ResNet 101 Equalized Histogram + Sharpen Features extracted using pre trained weights of VGG 19	Random forest Random forest	**96.94** **99.65**	**0.976** **0.9983**

## Data Availability

The data used for this study publicly available in https://data.mendeley.com/datasets/ftmp4cvtmb/1 (accessed on 16 January 2023).
